# Noradrenaline Protects Human Microglial Cells (HMC3) Against Apoptosis and DNA Damage Induced by LPS and Aβ_1-42_ Aggregates In Vitro

**DOI:** 10.3390/ijms252111399

**Published:** 2024-10-23

**Authors:** Julia Barczuk, Grzegorz Galita, Natalia Siwecka, Michał Golberg, Kamil Saramowicz, Zuzanna Granek, Wojciech Wiese, Ireneusz Majsterek, Wioletta Rozpędek-Kamińska

**Affiliations:** 1 Department of Clinical Chemistry and Biochemistry, Medical University of Lodz, 92-215 Lodz, Poland; julia.barczuk@stud.umed.lodz.pl (J.B.); grzegorz.galita@umed.lodz.pl (G.G.); natalia.siwecka@stud.umed.lodz.pl (N.S.); kamil.saramowicz@stud.umed.lodz.pl (K.S.); zuzanna.granek@stud.umed.lodz.pl (Z.G.); wojciech.wiese@stud.umed.lodz.pl (W.W.); ireneusz.majsterek@umed.lodz.pl (I.M.); 2 Department of Histology and Embryology, Medical University of Lodz, 92-215 Lodz, Poland; michal.golberg@umed.lodz.pl

**Keywords:** Alzheimer’s disease, noradrenaline, amyloid beta, LPS, neuroinflammation, microglia, HIF-1α, Bax, Bcl-2, caspase-3

## Abstract

Alzheimer’s disease (AD) is the most prevalent neurodegenerative disorder, characterized by the accumulation of amyloid-beta (Aβ) plaques and neuroinflammation. This study investigates the protective effects of noradrenaline (NA) on human microglial cells exposed to lipopolysaccharides (LPS) and Aβ aggregates—major contributors to inflammation and cellular damage in AD. The reduced Aβ aggregation in the HMC3 human microglial cells co-treated with Aβ and NA was confirmed by thioflavin T (ThT) assay, fluorescent ThT staining, and immunocytochemistry (ICC). The significantly increased viability of HMC3 cells after 48 h of incubation with NA at 50 µM, 25 µM, and 10 µM, exposed to IC50 LPS and IC50 Aβ, was confirmed by XTT and LDH assays. Moreover, we found that NA treatment at 25 μM and 50 μM concentrations in HMC3 cells exposed to IC50 LPS or IC50 Aβ results in an increased proliferation of HMC3 cells, their return to normal morphology, decreased levels of DNA damage, reduced caspase-3 activity, decreased expression of pro-apoptotic DDIT3 and BAX, and increased expression of anti-apoptotic BCL-2 genes and proteins, leading to enhanced cell survival, when compared to that of the HMC3 cells treated only with IC50 LPS or IC50 Aβ. Furthermore, we showed that NA induces the degradation of both extracellular and intracellular Aβ deposits and downregulates hypoxia-inducible factor 1α (HIF-1α), which is linked to impaired Aβ clearance and AD progression. These findings indicate that NA holds promise as a therapeutic target to address microglial dysfunction and potentially slow the progression of AD. Its neuroprotective effects, particularly in reducing inflammation and regulating microglial activity, warrant further investigation into its broader role in mitigating neuroinflammation and preserving microglial function in AD.

## 1. Introduction

Alzheimer’s disease (AD) is the most common neurodegenerative disorder, accounting for 60–80% of all dementia cases [1]. Although dementia is the seventh leading cause of death worldwide, its current treatment options are highly limited and do not reverse the disease’s progression. The hallmark of AD is the accumulation of extracellular amyloid beta (Aβ) plaques and intracellular neurofibrillary tangles (NFTs) containing tau-microtubule-associated protein (tau) [2]. Their accumulation in the neuronal bodies of noradrenergic neurons of locus coeruleus constitutes the “seed” of AD pathogenesis and contributes to neuronal cell death and AD progression [3]. However, due to the failure of numerous clinical trials targeted at reducing amyloid, it became clear that AD pathology is much more multifaceted, and new approaches to AD treatment are required [4].

Microglia, scavenger cells of the central nervous system (CNS), play a vital role in the innate immune response within a brain [5]. When triggered due to injury, chronic inflammation, extracellular amyloid, or intraneuronal phosphorylated tau, among others causes, ramified microglia undergo physiological rearmaments leading to its activation. Activated microglial cells exhibit two primary phenotypes, M1 and M2, of which M1 presents a pro-inflammatory phenotype, and M2 comprises phagocytic cells, executing an anti-inflammatory response [6]. Activated microglia, especially in interaction with astrocytes and neurons, constitute the major factor in the exacerbation of neuroinflammation, contributing to neurodegenerative diseases like AD [7].

Modulating microglia’s response on different levels is being studied for a better understanding of its role in inflammatory regulation. It has been observed that targeting reactive microglia via the mitogen-activated protein kinase (MAPK) signaling pathway reduced the neuroinflammatory behavior of the activated microglia, leading to the inhibition of neuronal degeneration and apoptosis [8]. Another commonly known agent which exerts an anti-inflammatory effect on activated microglial cells, although with an unclear mechanism of action, is noradrenaline (NA) [9]. NA has been observed to exert the greatest impact on AD neurodegeneration among all brain catecholamines; therefore, targeting NA activity may constitute a new potential approach to AD treatment [10]. NA involvement in AD was first noticed upon finding noradrenergic neuron deficits in the locus coeruleus of AD patients [11]. Since then, numerous studies have focused on analyzing how NA levels correlate with AD progression and a cognitive decline [12,13]. Currently, it is believed that NA’s impact on glial activation and immune homeostasis in the brain are the most significant aspects of the contribution of adrenergic signaling to neurodegeneration.

While NA is mainly suspected to have an anti-inflammatory effect, there are still some contradictory data regarding how noradrenergic signaling modulates microglial activity in the context of AD [14,15]. For that reason, it is vital to further characterize the pathways by which NA could modulate glial activity and innate immune responses in the brain [10]. Thus, the purpose of the proposed study is to assess how NA modulates microglial metabolism and the inflammatory response to Aβ in human microglial cells, as well as to determine whether NA impacts microglia survival in the pro-inflammatory environment.

## 2. Results

### 2.1. Confirmation of Aβ Aggregation

**Figure 1:**
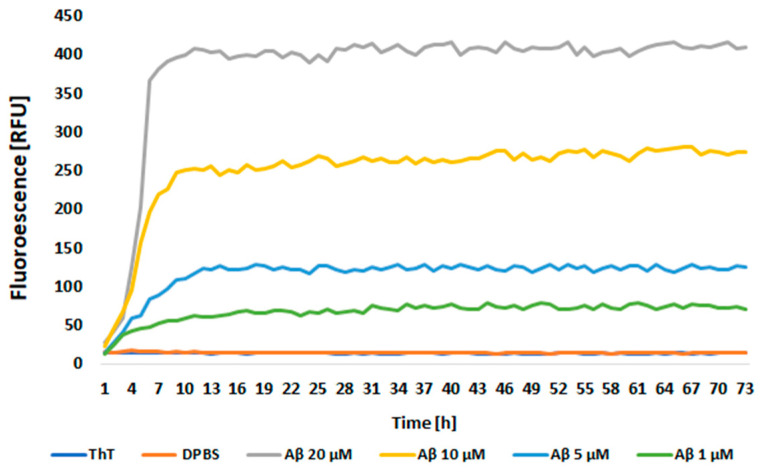
The thioflavin T (ThT) assay for the presence of beta-amyloid aggregates. The kinetic curves show the concentration-dependent increase in fluorescence induced by aggregated Aβ.

The presence of Aβ aggregates was confirmed by the thioflavin T (ThT) assay, which revealed an increase in fluorescence induced by aggregated Aβ in a concentration-dependent manner (Figure 1). The presence of Aβ deposits in the cell cultures was additionally validated by fluorescent ThT staining and immunocytochemistry (ICC; please see Section 2.5 and Section 2.6 for more details).

### 2.2. Analysis of the Cytotoxic Effect of the Noradrenaline on HMC3 Cells Exposed to LPS or Aβ

**Figure 2:**
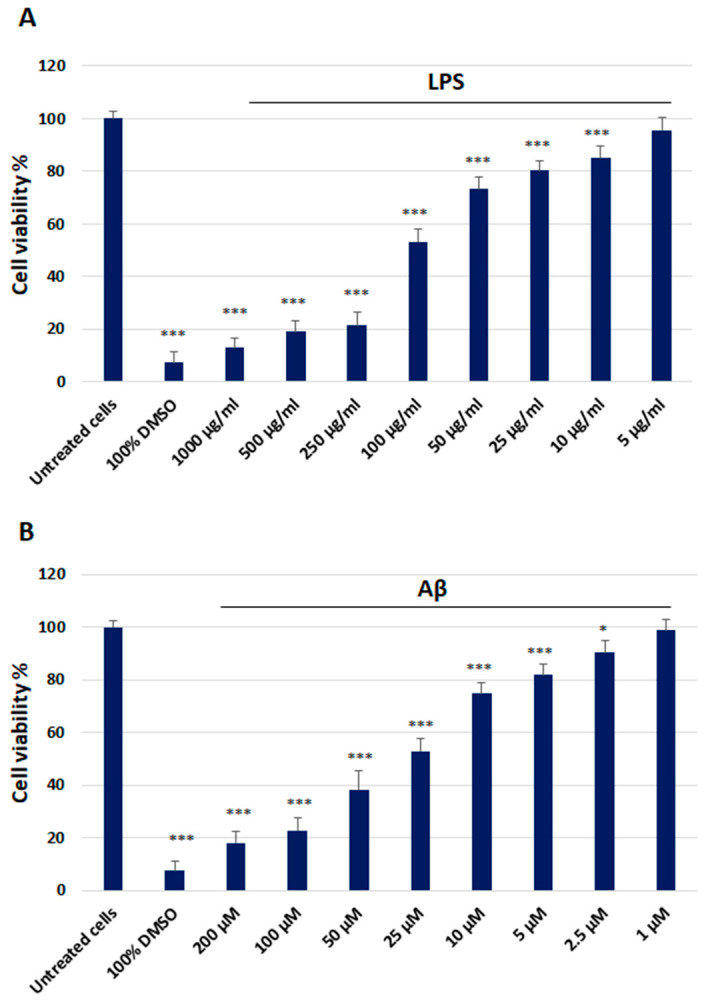
The cytotoxicity analysis of the lipopolysaccharide (1000 µg/mL–5 µg/mL) (**A**) and beta-amyloid (200 µM–1 µM) (**B**) in HMC3 cells assessed using the XTT Assay Kit. Cells were incubated with the indicated compounds for 48 h. The one-way ANOVA with Bonferroni post-hoc test was used in the statistical analysis. All the experiments were performed in triplicate, and the data are expressed as the mean ± SD, * *p* < 0.05, *** *p* < 0.001 vs. untreated cells.

The cytotoxicity of the LPS and Aβ, as well as the evaluation of the effect of NA on HMC3 cells exposed to LPS and Aβ, were measured using the 2,3-bis-(2-methoxy-4-nitro-5-sulfophenyl)-2H-tetrazolium-5-carboxanilide (XTT) colorimetric assay. Both LPS and Aβ evoked the cytotoxic effect on HMC3 cells, and the IC50 value for LPS was 101.28 µg/mL and for Aβ, it was 24.52 µM, respectively (Figure 2).

**Figure 3:**
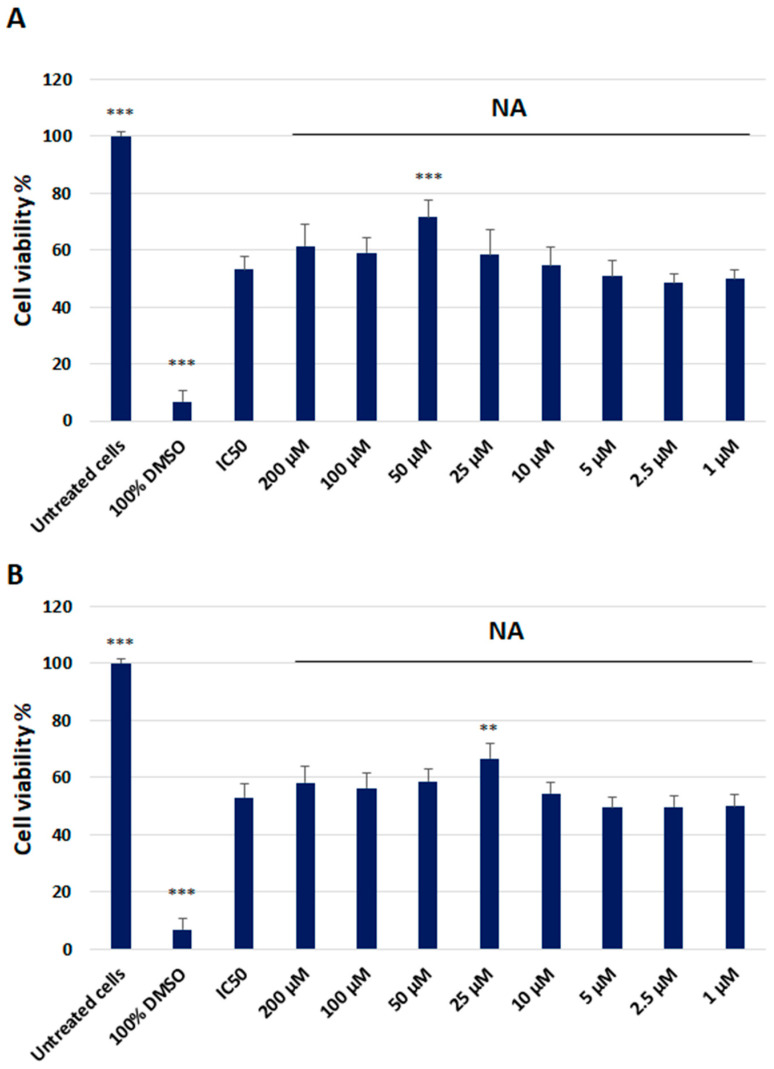
The cytotoxicity analysis of the noradrenaline (200 µM–1 µM) in HMC3 cells exposed to IC50 lipopolisacharide (µg/mL) (**A**) and IC50 amyloid beta (µM) (**B**), assessed using the XTT Assay Kit. Cells were incubated with the indicated compounds for 48 h. The one-way ANOVA with Bonferroni post-hoc test was used in the statistical analysis. All the experiments were performed in triplicate, and the data are expressed as the mean ± SD, ** *p* < 0.01, *** *p* < 0.001 vs. IC50.

NA at a concentration of 50 µM significantly increased the viability of HMC3 cells exposed to IC50 LPS. No significant effect toward HMC3 cells exposed to IC50 LPS was observed at any other of the applied concentrations of the NA. Moreover, 25 µM NA markedly increased HMC3 cell viability when exposed to IC50 Aβ. There was no effect on HMC3 cells exposed to IC50 Aβ or any other tested concentrations of the NA (Figure 3).

**Figure 4:**
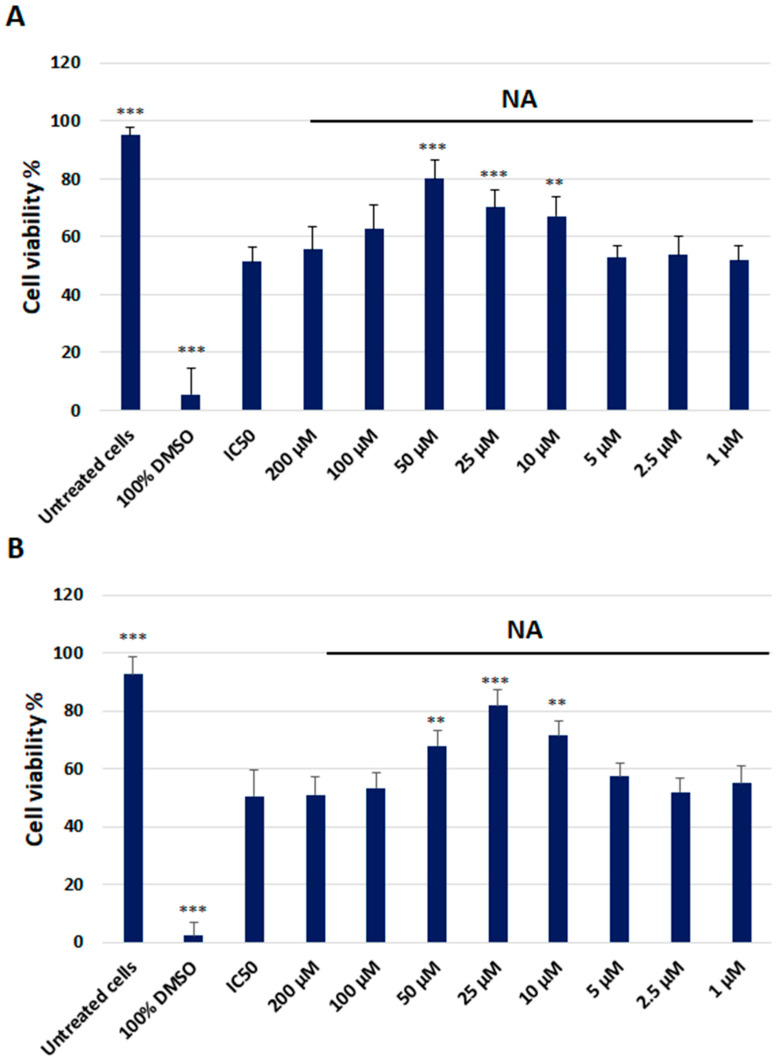
The cytotoxicity analysis of the noradrenaline (200 µM–1 µM) in HMC3 cells exposed to IC50 lipopolisacharide (µg/mL) (**A**) and IC50 amyloid beta (µM) (**B**), assessed using the Pierce LDH Cytotoxicity Assay Kit. Cells were incubated with the indicated compounds for 48 h. The one-way ANOVA with Bonferroni post-hoc test was used in the statistical analysis. All the experiments were performed in triplicate, and the data are expressed as the mean ± SD, ** *p* < 0.01, *** *p* < 0.001 vs. IC50.

The effect of NA on HMC3 line viability after LPS and Aβ treatment was also assessed using the Pierce LDH Cytotoxicity Assay Kit. NA at 50 µM, 25 µM, and 10 µM concentrations significantly increased the viability of HMC3 cells exposed to IC50 LPS. No significant effect toward HMC3 cells exposed to IC50 LPS was observed at any of the other applied concentrations of the NA. Moreover, 50 µM, 25 µM, and 10 µM NA markedly increased HMC3 cell viability when exposed to IC50 Aβ. There was no effect on HMC3 cells exposed to IC50 Aβ or any other tested concentrations of the NA (Figure 4).

### 2.3. Effect of the Noradrenaline on Morphological Changes in HMC3 Cells Exposed to LPS or Aβ

**Figure 5:**
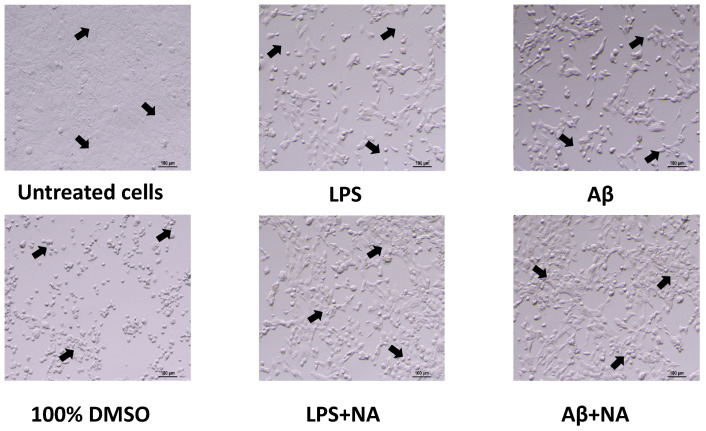
Effect of noradrenaline on the morphology of HMC3 cells exposed to LPS or Aβ after 24 h incubation. The main differences between groups are marked with arrows. Morphology images were captured using an inverted phase-contrast microscope in Nis elements D software ver. 5.30, with a magnification 20×. Abbreviations: LPS—lipolisacharide; Aβ—amyloid beta; NA—noradrenaline.

The effect of the NA on the morphological changes in HMC3 cells exposed to LPS or Aβ was assessed by phase-contrast microscopy. The normal morphology of the HMC3 cells was disturbed after their treatment with IC50 LPS or IC50 Aβ for 24 h, as compared to that of the untreated cells. The loosening of cell–cell contact and the acquisition of a more spindle-shaped morphology after treatment with IC50 LPS or IC50 Aβ within the HMC3 cells were observed. Moreover, IC50 LPS- or IC50 Aβ-treated HMC3 cells lost their capacity to attach to the culture vessels and became suspended. However, after treatment of the HMC3 cells with IC50 LPS or IC50 Aβ with the NA for 24 h, an increase in the proliferation of HMC3 cells, their return to normal morphology, and a decreased ability to detach from the culture vessel was observed (Figure 5).

### 2.4. Evaluation of the Genotoxicity of the Noradrenaline on HMC3 Cells Exposed to LPS or Aβ

**Figure 6:**
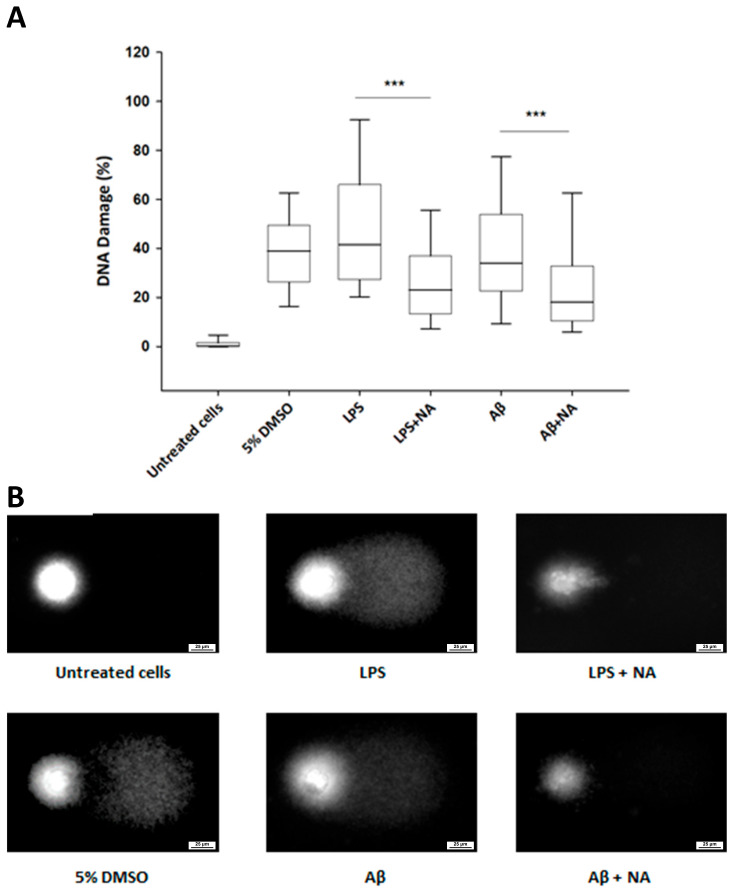
Evaluation of genotoxicity of the noradrenaline (µM) in HMC3 cells exposed to IC50 lipopolysaccharide (µg/mL) and IC50 amyloid beta (µM) (**A**), assessed using the alkaline version of the comet assay. The representatives of each group of comet assay images were captured using an inverted fluorescent microscope in Lucia Comet Assay software ver. 7.60. (**B**), The Kruskal–Wallis test was applied for the statistical analysis. All the experiments were conducted in triplicate, and the data are expressed as the median and interquartile range. *** *p* < 0.001 for all groups. Abbreviations: LPS—lipolisacharide; Aβ—amyloid beta; NA—noradrenaline.

To assess the level of DNA damage induced by IC50 LPS and IC50 Aβ, as well as to evaluate the effect of NA on HMC3 cells treated with IC50 LPS or IC50 Aβ, we used the alkaline version of the comet assay. The obtained results demonstrated that NA significantly decreased the level of DNA damage in the HMC3 cells induced by IC50 LPS or IC50 Aβ (Figure 6).

### 2.5. Thioflavin T-Stained Fluorescence Microscopy Imaging

**Figure 7:**
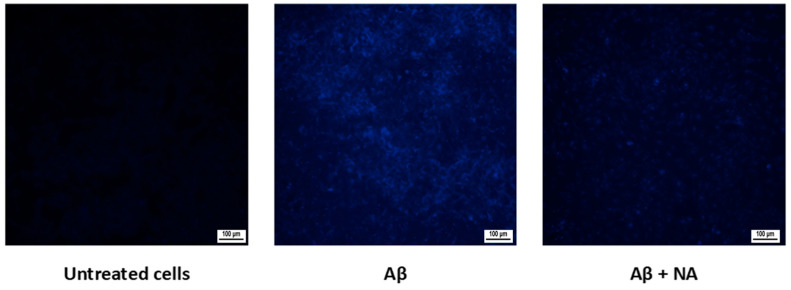
Fluorescence imaging of ThT-stained cells grown under different conditions: untreated HMC3 cells (control), HMC3 cells treated with amyloid β (Aβ), HMC3 cells treated with amyloid β and noradrenaline (Aβ + NA).

The presence of Aβ deposits in the cell cultures was evidenced by fluorescent ThT staining. HMC3 cells treated with Aβ showed an increased fluorescence compared to that of the untreated cells. Co-treatment with NA resulted in a decreased fluorescent signal, indicative of a reduced Aβ aggregation in the cells (Figure 7).

### 2.6. Immunocytochemistry

**Figure 8:**
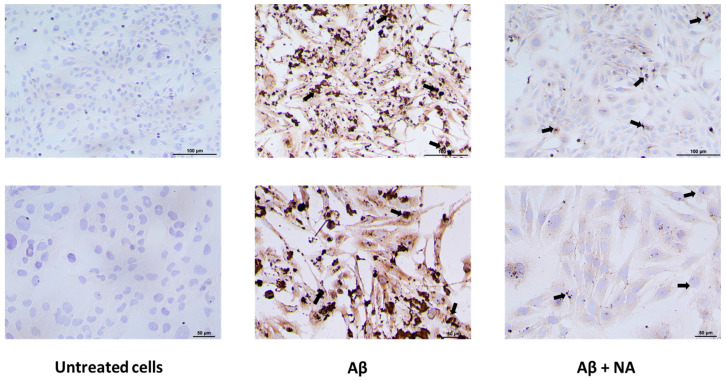
Immunocytochemical staining for amyloid β (Aβ) in cells grown under different condi-tions: untreated HMC3 cells, HMC3 cells treated with Aβ, HMC3 cells treated with amyloid β and noradrenaline (Aβ + NA). The main differences between groups are marked with arrows, with magnifications of 10× (upper row) and 20× (lower row). Cells were counterstained with hematoxylin. Representative images are shown.

The immunocytochemical staining for Aβ revealed the expression of both extracellular and intracellular aggregates in the HMC3 cells treated with Aβ in contrast to the results for untreated cells. The co-treatment with NA reduced the expression of both extracellular and intracellular Aβ deposits (Figure 8).

### 2.7. Assessment of the Level of Apoptosis in HMC3 Cells Exposed to LPS or Aβ

**Figure 9:**
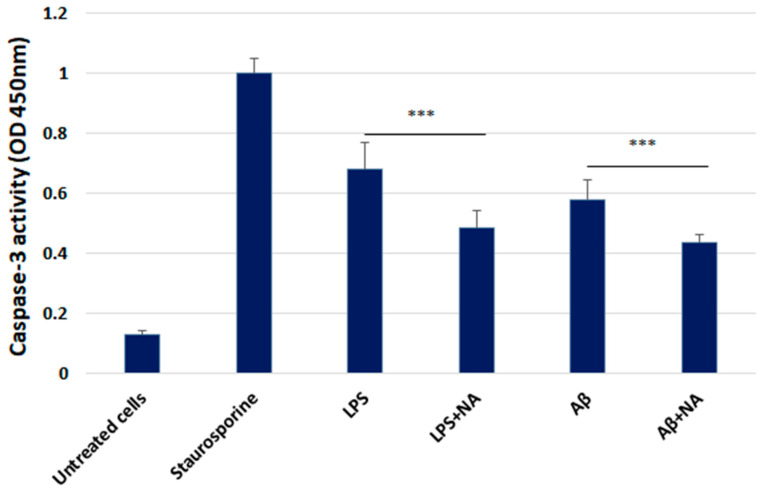
Colorimetric assessment of the level of caspase-3 in HMC3 cells exposed to IC50 lipopolysaccharide (µg/mL) and IC50 amyloid beta (µM) only or in the presence of noradrenaline. The one-way ANOVA with Bonferroni post-hoc test was used in the statistical analysis. All the experiments were performed in triplicate, and the data are expressed as the mean ± SD. *** *p* < 0.001 for all groups. Abbreviations: LPS—lipopolysaccharide; Aβ—amyloid beta; NA—noradrenaline.

The colorimetric caspase-3 assay was used to assess the caspase-3 activity level in the HMC3 cells treated with IC50 LPS or IC50 Aβ in the presence of NA. The obtained results demonstrated that 1 μM staurosporine evoked a significant increase in caspase-3 activity in HMC3 cells after 16 h of incubation in comparison with that of the HMC3 cells used as a negative control (untreated cells). NA evoked a significant decrease in caspase-3 activity in the HMC3 cells exposed to IC50 LPS or IC50 Aβ (Figure 9).

### 2.8. Assessment of the ATP Rate in HMC3 Cells Exposed to LPS or Aβ

**Figure 10:**
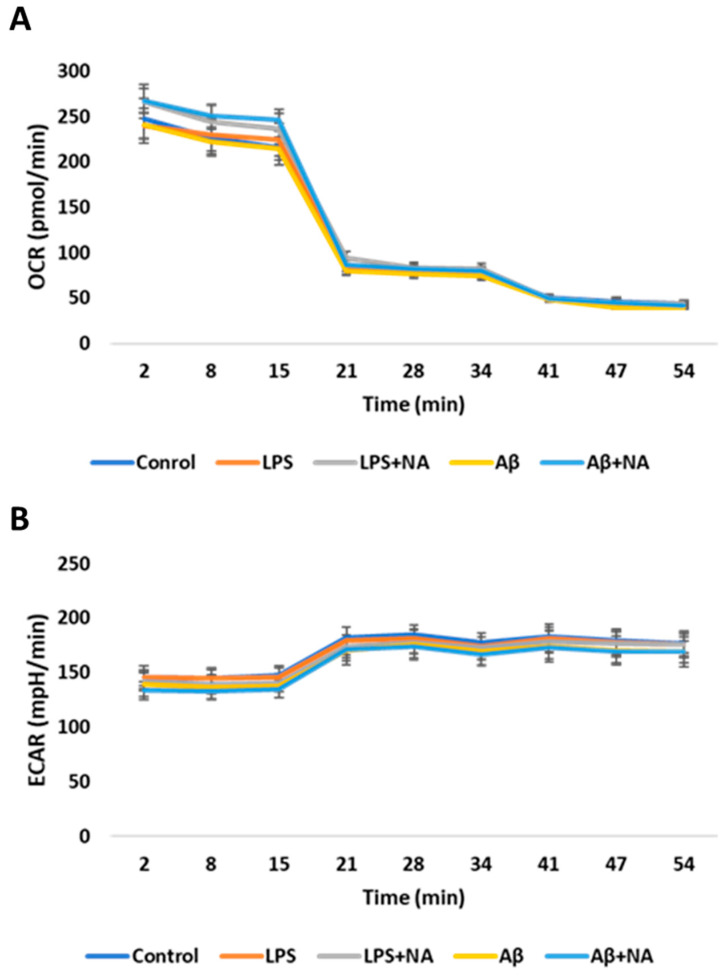
Measurement of oxygen consumption rate (OCR) (**A**) and extracellular acidification rate (ECAR) (**B**) in real-time by Seahorse ATP Rate assay in HMC3 cells exposed to IC50 lipopolysaccharide (µg/mL) and IC50 amyloid beta (µM) only or in the presence of noradrenaline. The one-way ANOVA with Bonferroni post-hoc test was used in the statistical analysis. All the experiments were performed in triplicate, and the data are expressed as mean ± SD. Abbreviations: LPS—lipopolysaccharide; Aβ—amyloid beta; NA—noradrenaline.

To assess the ATP rate in the HMC3 cells treated with the investigated compounds, the Seahorse ATP Rate Assay was used. The OCR and ECAR values of cells treated with IC50 LPS or IC50 Aβ did not significantly differ from the HMC3 cells treated in the same manner and in the presence of NA (Figure 10).

### 2.9. Evaluation of the mRNA Expression Level in HMC3 Cells Exposed to LPS or Aβ

**Figure 11:**
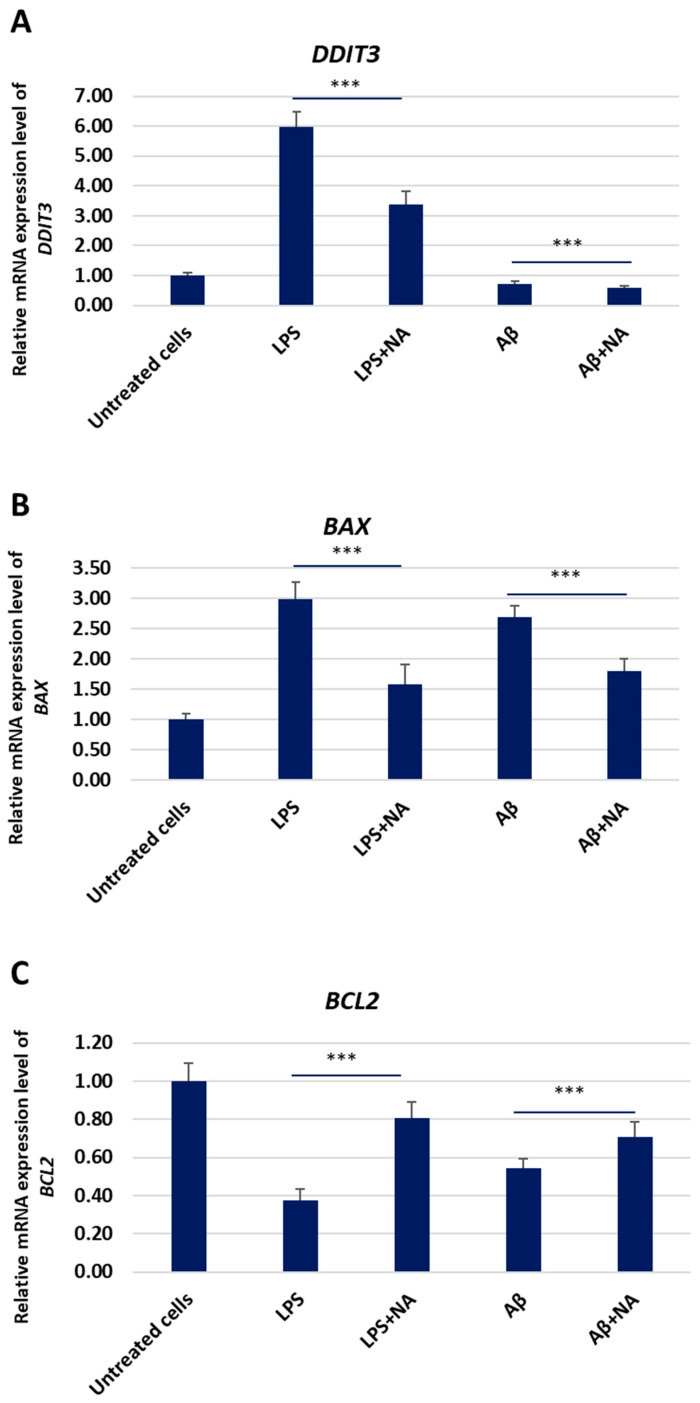
The mRNA expression levels of DDIT3 (**A**), BAX (**B**), and BCL2 (**C**) genes in HMC3 cells exposed to IC50 lipopolisacharide (µg/mL) and IC50 amyloid beta (µM) only or in the presence of noradrenaline. ACTB was used as a reference gene. The one-way ANOVA with Bonferroni post-hoc test was applied for the statistical analysis. All the experiments were performed in triplicate, and the data are expressed as the mean ± SD. *** *p* < 0.001 for all groups. Abbreviations: LPS—lipolisacharide; Aβ—amyloid beta; NA—noradrenaline.

The mRNA expression levels of *DDIT3*, *BAX,* and *BCL2* were assessed in the HMC3 cells treated only with IC50 LPS or IC50 Aβ, as well as co-treated with NA. There was a significant decrease in the mRNA expression level of pro-apoptotic genes such as *DDIT3* and *BAX* in the HMC3 cells in the presence NA as compared to that of the HMC3 cells treated only with IC50 LPS or IC50 Aβ. However, in the HMC3 cells in the presence of NA, a significant increase in the mRNA expression level of the anti-apoptotic gene *BCL-2* was demonstrated as compared to that in the HMC3 cells treated only with IC50 LPS or IC50 Aβ (Figure 11).

### 2.10. Evaluation of the of the Protein Expression Level in HMC3 Cells Exposed to LPS or Aβ

**Figure 12:**
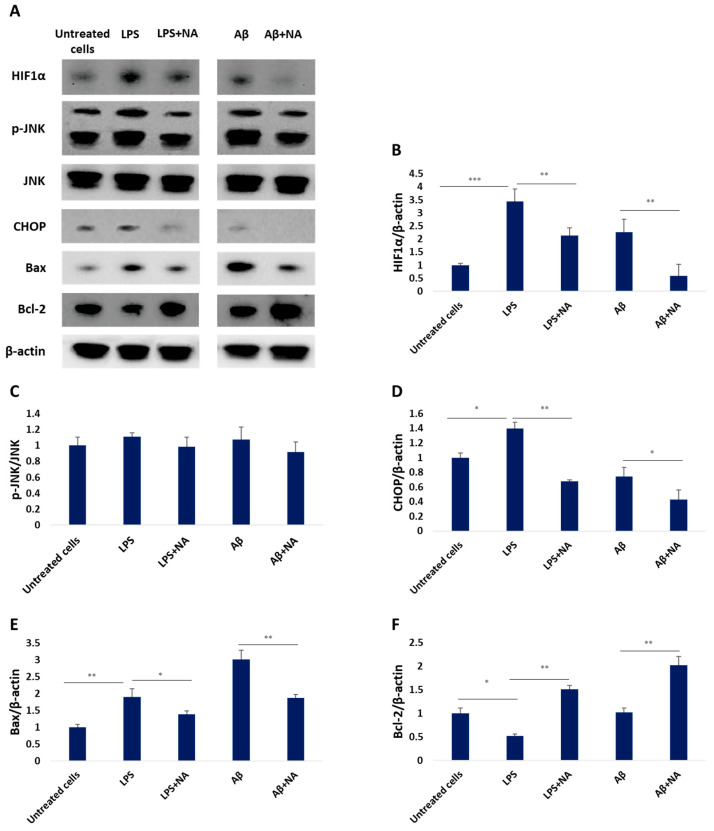
Western blot analysis (**A**) of the expression level of the proteins HIF-1α (**B**), p-JNK (**C**), CHOP (**D**), Bax (**E**), and Bcl-2 (**F**) in HMC3 cells exposed to IC50 lipopolysaccharide (µg/mL) and IC50 amyloid beta (µM) only or in presence of noradrenaline. β-actin was applied as a loading control. The one-way ANOVA with Bonferroni post-hoc test was used in the statistical analysis. All the experiments were conducted in triplicate, and the data are expressed as the mean ± SD. * *p* < 0.05, ** *p* < 0.01, *** *p* < 0.001 for all groups. Abbreviations: LPS—lipopolysaccharide; Aβ—amyloid beta; NA—noradrenaline.

The obtained results demonstrated that the level of HIF-1α protein was significantly upregulated by LPS and Aβ and downregulated upon co-treatment with NA. On the other hand, there was no significant difference in the level of p-JNK after LPS, Aβ, and NA exposure. Interestingly, treatment with LPS increased the level of pro-apoptotic CHOP factor, while treatment with Aβ decreased it, whereas co-treatment with NA further reduced CHOP expression. IC50 LPS or IC50 Aβ significantly increased the level of pro-apoptotic BAX protein in the HMC3 cells, and NA treatment reversed these changes. Moreover, the co-treatment with NA markedly increased the level of anti-apoptotic protein BCL-2 (Figure 12).

## 3. Discussion

To date, the vast majority of studies addressing the impact of NA on microglial dynamics are based on observations from rodent models. There is robust evidence of key discrepancies between rodent and human microglia responses, arguing for greater translatability and clinical relevance of human microglia cultures [16]. Therefore, in the present study, we incorporated the HMC3 cell line, which accurately reflects the phenotypical and morphological properties of the cortical microglia in the human brain [17].

While microglial activation has traditionally been linked to the M1 pro-inflammatory phenotype and the secretion of cytokines that promote neurodegeneration, recent studies suggest a broader role for microglial dysfunction in AD. This includes senescence, mitochondrial dysfunction, and finally, microglial apoptosis, all of which disrupt the proper clearance and surveillance functions of microglia in the CNS [18,19]. Our qualificative assessment of microglial morphology demonstrated a switch from the resting microglial state to the hypertrophied intermediately activated state, suggestive of enhanced Aβ phagocytosis. On the other hand, upon NA treatment, activated HMC3 cells tend to adopt a more ramified resting state [20].

Microglia cells are the most efficient scavengers of Aβ oligomers and fibrils in the brain, showing an uptake 3–4 times higher than that of other cell types [20]. As the amyloid burden increases, activated microglia struggle to clear the internalized Aβ peptides [21,22]. This failure leads to the accumulation of Aβ in the lysosomes, ultimately resulting in microglial cell death in vitro and in vivo. Dying microglia release accumulated Aβ into the extracellular space, which promotes Aβ plaque growth [22]. Furthermore, considering the migratory properties of microglia, prior to death, they can transport Aβ toward previously unaffected brain tissue, contributing to the propagation of a pathology [23]. Our fluorescent ThT staining revealed that a co-treatment of Aβ with NA resulted in the reduced unspecific Aβ aggregation in cells compared to that of HMC3 cells treated only with Aβ. Moreover, our immunocytochemical staining for Aβ revealed that co-treatment with NA reduced the specific expression of both extracellular and intracellular Aβ deposits compared to that of cells treated only with Aβ. These results constitute evidence of a clear effect of NA on Aβ aggregation, which can be explained by the recent molecular dynamics simulation study revealing the molecular mechanism by which NA molecules inhibit Aβ aggregation and disaggregate Aβ protofibrils. It was proved that NA molecules significantly reduce the interpeptide β-sheet content and suppress the formation of the β-hairpin-containing structure, leading to a more disordered coil-rich Aβ dimer. Also, NA molecules destabilize Aβ protofibril by forming H-bonds with the particular residues. Moreover, it was noted that the hydrophobic interactions, aromatic stacking, hydrogen bonding, and cation-π interactions collaboratively enhance the binding of NA molecules to Aβ peptides [24].

In a study by Baik et al., signals of cleaved caspase-3, an apoptosis marker, were detected near Aβ plaques and inside activated microglia in a mouse AD model, suggesting that Aβ-induced microglial cell death occurs via apoptosis [22]. Furthermore, the presence of elevated levels of cleaved caspase-3 was found within the reactive microglia in the frontal cortex of AD patients [25]. Intriguingly, in a study by Burguillos et al., an increase in caspase-3 activity in the microglia was shown to mediate LPS-induced neurotoxicity by promoting microglial proinflammatory activation. In the same study, co-culturing murine BV2 microglia with MN9D neurons revealed that LPS treatment induces neuronal cell death, while caspase-3/7 knockdown in the microglia cells prevents LPS-induced neurodegeneration [25]. Collectively, caspase-3 and microglial apoptosis may be potential therapeutic targets against the spread of Aβ plaques and the microglia-mediated neurotoxicity in AD [22,25]. In our study, we observed that treating human microglia cells with LPS or Aβ indeed leads to a marked increase in cleaved caspase-3 activity and a dose-dependent reduction in cell viability. Since Aβ aggregates are known to induce mitochondrial dysfunction, to perform a more objective, mitochondrial-independent analysis of cytotoxicity and cell viability, we additionally employed the LDH assay. The LDH assay specifically measures cell membrane integrity, which renders it more sensitive in detecting necrosis or late apoptosis than XTT. We observed significant LDH release after 48 h of Aβ aggregate treatment, in line with the results of previous papers [26]. Interestingly, NA treatment considerably reduced LDH leakage, further confirming the role of NA in rescuing human microglia from Aβ-induced toxicity.

In addition, treatment of human microglia cells with LPS or Aβ increased the expression of pro-apoptotic Bax, while decreasing anti-apoptotic Bcl-2 at both the mRNA and protein levels, which is essential for potential loss of the mitochondrial membrane and the proapoptotic release of cytochrome c [27]. This mitochondrial apoptosis pathway was previously found to be induced by Aβ aggregates in primary murine microglia, i.e., the N13 and BV2 microglial cell lines [27,28,29,30]. We showed that NA treatment significantly increased cell viability and the expression of anti-apoptotic Bcl-2, while reducing the expression of pro-apoptotic Bax and caspase-3 activity in both the LPS and Aβ treated groups. Therefore, NA exerts a protective effect on human microglia, potentially contributing to the alleviation of AD-associated microglia neurotoxicity and the spreading of Aβ pathology.

In addition to the apoptosis, another mechanism impairing protective Aβ clearance is related to the upregulation of the hypoxia-inducible factor 1α (HIF-1α) in Aβ plaque-associated microglia [31]. Enhanced expression of HIF-1α in Aβ plaque-associated microglia has been confirmed in various in vivo AD models, and activation of HIF-1α signaling has been proposed as a detrimental modulator of AD pathology, as enhanced or inhibited HIF-1α signaling occurred concurrently with enhanced or attenuated Aβ deposition [32]. It has been previously demonstrated that Aβ or LPS treatment resulted in the dramatically increased expression of HIF-1α in primary murine microglial cells [33]. We also observed a significant increase in HIF-1α protein expression in human microglia cells treated with Aβ or LPS. Interestingly, NA treatment notably reduced HIF-1α expression, suggesting its potential alleviating impact of HIF-1α-mediated microglial reprogramming. In macrophages, proinflammatory stimulus, like LPS, suppresses cell proliferation via the Myc-dependent pathway, while activating HIF-1α-dependent transcriptional reprograming to maintain the heightened glycolysis of M1 macrophages. Switching between Myc-dependent and HIF-1α-dependent transcriptional pathways ensures that proinflammatory M1-macrophages will maintain sufficient metabolic capacity to sustain their effector function, while limiting cell proliferation-associated energy loss [34]. A similar metabolism-shifting mechanism was described by Baik et al., in which HIF-1α activation was found to be required for the metabolic switching of the microglia from mitochondrial oxidative phosphorylation (OXPHOS) to anaerobic glycolysis, which cannot provide microglia with sufficient energy (ATP) to remove Aβ, thereby impairing their phagocytic capacity and Aβ clearance [33]. However, a study by March-Diaz, Rosana et al. showed that the OXPHOS gene set was paradoxically enriched upon activation of the HIF-1α pathway, and the oxygen consumption rate (OCR) was increased in primary mouse microglia treated with Aβ oligomers [31]. OCR reflects mitochondrial oxidative respiration, which was previously found to be critical for phagocytosis of microglial Aβ [33]. Considering these conflicting reports, we conducted a Seahorse analysis to address the impact of LPS, Aβ, and NA on mitochondrial respiration. Surprisingly, our Seahorse experiment demonstrated very subtle differences in OCR and extracellular acidification rate (ECAR) in all experimental groups. Therefore, further research, especially on human microglia cells, is warranted to elucidate the role of mitochondrial metabolism in AD preclinical models.

LPS and Aβ can directly stimulate NADPH oxidase-mediated ROS production in microglia, leading to oxidative DNA damage, which has been shown in AD mouse models to promote hippocampal neurodegeneration [35,36,37,38]. Moreover, a recent study revealed that microglia in the brains of AD patients express markers of DNA damage, which contributes to premature microglial senescence and impairs Aβ phagocytosis and clearance, thereby accelerating disease progression [38]. Our alkaline comet assay demonstrated that both Aβ and LPS robustly induce DNA damage in human microglia cells. Interestingly, NA treatment significantly reduced the rate of DNA damage in both Aβ and LPS-treated microglia cells, highlighting the gene-protective properties of NA. Neurotransmitters such as catecholamines, in this study represented by NA, play an established role in inhibiting ROS-associated DNA cleavage. Catecholamines display a phenol structure able to scavenge ROS by hydrogen atom transfer from a phenolic hydroxyl group to ROS [39].

While this study has provided some novel data on the impact of NA on microglia function in AD in vitro, several limitations are worth addressing. Firstly, the conditions of cell culture treatment, including the concentrations of Aβ and NA and the incubation time, may not accurately correspond to conditions observed in vivo [40,41]. Secondly, considering the highly complex dynamics of glia-glia and neuron-glia interactions, microglia in vitro may exhibit more artificial responses to stimuli due to the absence of modulating signals from other cells of the brain microenvironment [42,43]. Finally, the heterogeneity of AD-associated microglia and the molecular events associated with aging and microglial senescence should be taken into account to fully elucidate the effects of NA on microglial functioning [44]. Therefore, further studies involving in vivo or organ-on-chip models and high-throughput techniques are needed.

## 4. Materials and Methods

### 4.1. Cell Culture

The experiments were carried out using an in vitro cellular model with the human microglial cell line HMC3, which was commercially obtained and originally derived from brain tissue. HMC3 cells were procured from ATCC (Manassas, VA, USA). The cultures were maintained under standard conditions (37 °C, 5% CO_2_, 95% humidity), according to the vendor’s instructions. The cells were cultured in Eagle’s Minimum Essential Medium (EMEM), supplemented with 10% fetal bovine serum (FBS) (ATCC, Manassas, VA, USA) and 1% penicillin/streptomycin (P/S) (ScienCell Research Laboratories, San Diego, CA, USA). When the cultures reached 90–95% confluency, typically every 3–4 days, they were passaged using a 0.25% trypsin/EDTA (T/E) solution (Innoprot, Leioa, Bizkaia, Spain) for cell detachment.

### 4.2. Aggregation of Aβ

Aβ stock powder (Sigma Aldrich, St. Louis, MO, USA) was dissolved in DMSO to prepare the stock solutions. For the experiments, the stock solution was further diluted in a cold cell culture medium and incubated at 4 °C for 24 h. The sample was sonicated for 20 min before use. To confirm the presence of aggregation, a ThT assay was performed. ThT (Sigma Aldrich, St. Louis, MO, USA) was diluted in PBS pH 7.4 and added to the wells of the 96-well plate at 25 μM. Then, Aβ was added to the appropriate wells at a 50-1 μM concentration range before mixing. The fluorescence was measured at one-hour intervals for 72 h using a Synergy HT microplate reader (BioTek, Hong Kong, China).

### 4.3. Cytotoxicity Analysis

#### 4.3.1. XTT Colorimetric Assay

Cytotoxicity was assessed using the XTT colorimetric assay (Thermo Scientific, Waltham, MA, USA), which detects cellular metabolic activity. In this assay, the yellow tetrazolium salt XTT is reduced by dehydrogenase enzymes in metabolically active cells, producing a highly colored formazan dye. All experiments were performed in triplicate, with similar results. HMC3 cells were seeded into 96-well plates at a density of 5 × 10^3^ cells per well and cultured in 100 µL of complete EMEM medium for 24 h. Subsequently, the cells were treated with 100 µL of medium containing lipopolysaccharide (LPS) isolated from Escherichia coli O111:B4 compound at various concentrations (5 µg/mL, 10 µg/mL, 25 µg/mL, 50 µg/mL, 100 µg/mL, 250 µg/mL, 500 µg/mL, 1000 µg/mL) or Aβ at concentrations ranging from 1 µM to 200 µM. Once the IC50 value for each compound was determined, the cells were incubated with LPS or Aβ at their respective IC50 concentrations in the presence of NA, tested over a concentration range of 1 µM to 200 µM. Cells cultured in complete medium alone served as the negative control, while cells exposed to 100% DMSO served as the positive control. After 48 h of incubation, 25 µL of the XTT/PMS mixture was added to each well, as per the manufacturer’s instructions. After a 2 h incubation at 37 °C in a 5% CO_2_ incubator, the absorbance was measured at 450 nm using a Synergy HT spectrophotometer (BioTek, Hong Kong, China).

#### 4.3.2. Lactate Dehydrogenase Assay

The release of LDH into the culture medium was assessed using a Pierce™ LDH Cytotoxicity Assay Kit (Thermo Scientific, Waltham, MA, USA). Extracellular LDH in the culture media is quantified by a coupled enzymatic reaction with LDH, which catalyzes the conversion of lactate to pyruvate via the NAD^+^ reduction to NADH. Subsequently, diaphorase uses NADH to reduce tetrazolium salt to a red formazan product, measured spectrophotometrically. All experiments were performed in triplicate, with similar results. HMC3 cells were seeded in 96-well plates (5 × 10^3^/well) and cultured in 100 μL of complete EMEM growth medium for 24 h. After cell adhesion, the cells were treated with the IC50 LPS or IC50 Aβ. The cells were then treated with NA (50 μM for LPS and 25 μM for Aβ). After incubation, 50 μL of supernatant from each sample were transferred into new 96-well plates, and subsequently, 50 μL of the reaction mixture was added to each well. Following a 30 min incubation in the dark at room temperature (RT), the reaction was stopped via adding 50 μL of stop solution. Absorbance was measured at wavelengths of 490 nm and 680 nm (background) using the Synergy HT (BioTek, Hong Kong, China) spectrophotometer.

#### 4.3.3. Analysis of Morphological Changes

To evaluate the morphological changes of the HMC3 cells exposed to LPS or Aβ, after their treatment with NA, phase-contrast microscopy was used. The HMC3 cells were seeded on T-75 culture vessels (1 × 10^6^ cells) and cultured in a complete EMEM growth medium for 24 h. After cell adhesion, the cells were treated for 48 h with IC50 LPS or IC50 Aβ, and then they were treated with NA. Untreated cells cultured in a complete EMEM growth medium were used as a negative control, whereas cells incubated with 100% DMSO comprised a positive control. The morphological changes in the HMC3 cells was assessed using a Nikon (Nikon, Tokyo, Japan) inverted microscope at 20× magnification.

### 4.4. Genotoxicity Analysis

Genotoxicity was assessed using the alkaline comet assay (ACA), a highly sensitive method for detecting DNA strand breaks in cells. All experiments were conducted in triplicate, with similar results. The assays were set up in 6-well plates, with each well containing 2 × 10^5^ cells in 2 mL of complete EMEM medium. The cells were then treated with LPS or Aβ at their respective IC50 concentrations, either alone or in combination with NA (50 µM for LPS, 25 µM for Aβ). Cells suspended in 5% DMSO (Sigma-Aldrich, St. Louis, MO, USA) served as a positive control, while those in 2 mL of complete EMEM medium were used as a negative control. After 24 h of incubation, the cells were suspended in 0.37% low melting point (LMP) agarose (Sigma-Aldrich, St. Louis, MO, USA) and placed on microscope slides pre-coated with normal melting point (NMP) agarose (Sigma-Aldrich, St. Louis, MO, USA). The slides were then incubated in a lysis buffer (2.5 M NaCl, 10 mM Tris, 100 mM EDTA) containing 1% Triton X-100 (Sigma-Aldrich) at 4 °C for 60 min. Following lysis, the slides were incubated for 20 min in a development buffer (300 mM NaOH, 1 mM EDTA) at 4 °C, and electrophoresis was performed at 32 mA and 17 V for 20 min at 4 °C in an electrophoretic buffer (30 mM NaOH, 1 mM EDTA). After electrophoresis, the slides were rinsed three times with distilled water and air-dried at room temperature. The DNA damage was visualized by staining with DAPI and examination under a fluorescence microscope, with the percentage of DNA in the comet tail used to quantify the extent of DNA damage. The DNA tail was assessed using Lucia Comet Assay software ver. 7.60 (Laboratory Imaging s.r.o., Prague, Czech Republic).

### 4.5. Fluorescent Amyloid Detection

Cells were fixated in 10% neutral buffered formalin (NBF) (Sigma-Aldrich, St. Louis, MO, USA) for 1 h before staining. For amyloid fluorescence, cells were stained with 1% Thioflavin T (Sigma-Aldrich, St. Louis, MO, USA) water solution, according to Vassar and Culling’s method [45]. Amyloid fluorescence was observed under a fluorescent, inverted microscope (Nikon, Tokyo, Japan, software: NIS-Elements Advanced Research software, version 5.42, DAPI-5060C 1853394-3).

### 4.6. Immunocytochemistry (ICC) for β-Amyloid

A total of 10% of the NBF fixed cells were washed with PBS before the antigen retrial (AR) step. For AR cell culture, the plate’s wells were filled with high pH retrieval buffer (EnVision FLEX, Tris/EDTA pH 9, Dako, Hovedstaden, Denmark) and heated for 20 min at 95 °C. The cooled cells were washed with TBST for 5 min. The examined cells were treated with 3% hydrogen peroxidase solution for 5 min, then washed with TBST. Subsequently, the cells were treated with anti-Amyloid beta antibodies (1:200, β-Amyloid (D54D2) XP^®^ Rabbit mAb, #8243, Cell Signaling, Danvers, MA, USA) for 1 h in RT. Next, the cells were incubated with secondary antibodies conjoined with HRP (EnVision FLEX/HRP, Dako) for 20 min at RT. Incubation with DAB (3,3-diaminobenzidine, EnVision FLEX DAB, Dako) for 10 min at RT was used to obtain a chromatic product. Counterstaining for 5 min at RT with hematoxylin was employed. ICC amyloid-beta detection results were observed under an inverted microscope (Nikon, Tokyo, Japan, software: NIS-Elements Advanced Research software, version 5.42).

### 4.7. Apoptosis Analysis

Caspase-3 activity in the HMC3 cells was measured using a colorimetric caspase-3 assay kit (Abcam, Cambridge, UK), following the manufacturer’s instructions, which detects caspase-3 activity in cell lysates. Each experiment was repeated three times, with similar results. HMC3 cells were plated in 6-well plates at a density of 5 × 10^5^ cells per well and cultured in complete EMEM medium for 24 h. Following this, the cells were treated with LPS or Aβ at their respective IC50 concentrations, either alone or in combination with NA (50 µM for LPS, 25 µM for Aβ) for 48 h. Cells exposed to 1 µM staurosporine (Sigma-Aldrich, St. Louis, MO, USA) for 16 h served as a positive control, while cells cultured in EMEM alone for 48 h were used as a negative control. After treatment, the HMC3 cells were washed with 1X DPBS (Sigma-Aldrich, St. Louis, MO, USA) and detached using 0.25% trypsin/EDTA solution (Innoprot, Leioa, Bizkaia, Spain). The cell suspension was centrifuged at 1000 rpm for 5 min at room temperature, and the supernatant was discarded. The cells were then resuspended in complete EMEM, counted, centrifuged again for 5 min at 1000 rpm, and the pellet containing 1 × 10^6^ cells was resuspended in 50 µL of cold Cell Lysis Buffer. After 10 min of incubation on ice, the cell suspension was centrifuged at 10,000× *g* for 1 min, and the supernatants were transferred to fresh tubes. The protein concentration was determined using a standard Bradford assay, with BSA as the standard. For the caspase-3 activity assay, 100 µg of protein from each cell lysate was mixed with 2X Reaction Buffer containing 10 mM DTT, followed by the addition of DEVD-pNA substrate (final concentration 200 µM). The samples were incubated at 37 °C for 2 h, and the absorbance of the released p-NA was measured at 405 nm using a Synergy HT spectrophotometer (BioTek, Hong Kong, China).

### 4.8. Seahorse ATP Rate Assay

To investigate the effect of NA on cellular respiration—specifically oxidative phosphorylation and glycolysis—under conditions of LPS or Aβ toxicity, the Seahorse XFp Real-Time ATP Rate Assay (Agilent Technologies, Santa Clara, CA, USA) was used. This assay evaluates cellular metabolic activity by measuring both the oxygen consumption rate (OCR) and the extracellular acidification rate (ECAR). The cells were seeded into Seahorse XF HS Miniplates (Agilent Technologies, Santa Clara, CA, USA) and treated with the compounds, as previously described, while the untreated cells were used as controls. The day prior to the assay, the Seahorse XFp Sensor Cartridge was hydrated overnight in a non-CO_2_ incubator, following the manufacturer’s instructions. On the day of the assay, the cartridge was rehydrated with Seahorse XF Calibrant (Agilent Technologies, Santa Clara, CA, USA) and incubated for an additional 45 min. The cell culture medium was then removed from each well, and the cells were washed with prewarmed assay medium (pH 7.4), containing Seahorse XF DMEM, 10 mM glucose, 1 mM sodium pyruvate, and 2 mM L-glutamine (Agilent Technologies, Santa Clara, CA, USA), as per the manufacturer’s protocol. Afterward, the cells were incubated at 37 °C in a non-CO_2_ incubator for 45 min to allow the medium to reach the correct temperature and pH before the measurements were obtained. Inhibitors of ATP synthase (oligomycin, 2.5 µM) and mitochondrial complex I/III (rotenone/antimycin A, 0.5 µM) were prepared and loaded into the designated ports of the cartridge, as specified by the manufacturer. The analysis was conducted using the Seahorse XF HS Mini Analyzer (Agilent Technologies, Santa Clara, CA, USA). Three baseline measurements were obtained prior to any injections, followed by three additional measurements after the injection of each compound.

### 4.9. Analysis of the Expression of the ER Stress-Related Genes

Total RNA was extracted from the HMC3 cells using the PureLink RNA Mini Kit (Thermo Fisher Scientific, Waltham, MA, USA), following the manufacturer’s instructions. The isolated RNA was then reverse transcribed into cDNA at a final concentration of 100 ng using GoScript™ Reverse Transcriptase (Promega, Madison, WI, USA), as per the manufacturer’s protocol. To examine gene expression profiles associated with ER stress, a TaqMan Gene Expression Assay was conducted. The expression levels of the genes, including *DDIT3* (Hs01090850_m1), *BAX* (Hs00180269_m1), and *BCL2* (Hs00608023_m1), were measured, with *ACTB* (Hs99999903_m1) used as the reference gene (all genes are listed in Table 1). For the qPCR analysis, a total reaction volume of 10 µL was prepared, consisting of 1 µL cDNA, 1 µL primers, 2 µL 5x HOT FIREPol^®^ Probe qPCR Mix (Solis BioDyne, Tartu, Estonia), and 6 µL nuclease-free water. The reaction conditions followed those recommended by the manufacturer’s guidelines, including enzyme activation at 95 °C for 15 min, followed by 40 cycles of denaturation at 95 °C for 10 s, and annealing/extension at 60 °C for 60 s. The gene expression analysis was performed using the Bio-Rad CFX96 system (Bio-Rad, Hercules, CA, USA).

### 4.10. Western Blot Analysis

Western blot analysis was conducted to assess the effect of noradrenaline on the expression of specific proteins in cells treated with LPS or Aβ. The cells were treated with the compounds as described above, except for the control cells that remained untreated. After treatment, the cells were collected, and the total protein was extracted using the Minute™ Total Protein Extraction Kit (Invent Biotechnologies, Plymouth, MN, USA). The protein concentrations in the samples were measured and normalized using the Pierce™ BCA Protein Assay (Thermo Scientific, Waltham, MA, USA). The protein samples were denatured at 70 °C for 10 min, loaded onto gel wells, and then underwent electrophoresis for 50 min using the NuPage™/XCell SureLock™ system (Invitrogen, Waltham, MA, USA). The proteins were then transferred to a PVDF membrane (Invitrogen, Waltham, MA, USA) for 1 h. Afterward, the membrane was blocked for 1 h in a solution of 5% BSA or skim milk (BioShop Canada, Burlington, ON, Canada) in 1X TBST (Thermo Scientific Chemicals, Waltham, MA, USA), with BSA used for the phosphoproteins and skim milk for the non-phosphoproteins. The membranes were incubated overnight with primary monoclonal antibodies, depending on the experiment, targeting proteins such as HIF-1α, p-JNK, JNK, CHOP, Bax, and Bcl-2 (1:1000; Cell Signaling Technology, Danvers, MA, USA). β-Actin was used as the loading control (all antibodies are listed in Table 2). The following day, the membranes were washed three times with 1X TBST and incubated with secondary HRP-linked antibodies (1:5000; Cell Signaling Technology, Danvers, MA, USA). After a final wash in TBST, the membrane was incubated for 5 min with SuperSignal™ West Pico Chemiluminescent Substrate (Thermo Scientific, Waltham, MA, USA) in the dark, and the protein bands were detected using enhanced chemiluminescence (ECL) with the ChemiDoc™ Imaging System (Bio-Rad, Hercules, CA, USA). Densitometry analysis was performed using NIS-Elements Advanced Research software version 5.42 (Nikon, Tokyo, Japan).

### 4.11. Statistical Analysis

Statistical analysis was carried out using Statistica 13.3 software (StatSoft, Tulsa, OK, USA). The Shapiro–Wilk test was used to assess the normality of the data distribution, and Levene’s test was used to evaluate variance homogeneity. For all datasets, except those from the genotoxicity analysis, the data were found to be normally distributed and homogenous. Consequently, comparisons between two groups were made using the Student’s *t*-test, while comparisons among multiple groups were performed using one-way ANOVA, followed by Bonferroni post hoc tests. In the case of the genotoxicity assay, the data were analyzed using the Kruskal–Wallis test due to a non-normal distribution. All experiments were conducted in triplicate, using cells of the same passage number. Data in the graphs are presented as the mean ± SD (unless otherwise specified), and statistically significant differences are indicated as follows: * *p* < 0.05, ** *p* < 0.01, *** *p* < 0.001.

## 5. Conclusions

In summary, our data support the idea that NA protects human microglial cells against apoptosis and DNA damage induced by LPS and Aβ aggregates in vitro. We proved that NA treatment significantly increases cell viability and the expression of anti-apoptotic Bcl-2, while reducing the expression of pro-apoptotic Bax, CHOP, HIF-1α, and caspase-3 activity in both the LPS- and Aβ-treated groups. Therefore, NA exerts a protective effect on human microglia, potentially contributing to alleviating AD-associated microglia neurotoxicity and the spreading of Aβ pathology. In addition, NA treatment significantly reduces the rate of DNA damage in both Aβ- and LPS-treated microglia cells, highlighting the gene-protective properties of NA. As accumulating evidence indicates microglial dysfunction and apoptosis as the crucial molecular events contributing to neurodegeneration in AD, targeting NA as a regulator of microglial activity may constitute a new potential approach to AD treatment. Further studies are needed in order to confirm the advantages of the use of NA in AD, which may ultimately provide an effective disease-modifying strategy against AD.

## Figures and Tables

**Table 1:** The ID numbers of the applied TaqManTM Gene Expression Assays.

Gene Name	Encoded Protein	Assay ID
*DDIT3*	CHOP	Hs01090850_m1
*BAX*	Bax	Hs00180269_m1
*BCL2*	Bcl-2	Hs00608023_m1
*ACTB*	β-actin	Hs99999903_m1

**Table 2:** The ID numbers of the applied primary antibodies obtained from the Cell Signaling Technology.

Antibody Name	Dilution	Catalog No.
HIF-1α (D1S7W) XP^®^ Rabbit mAb	1:1000	#36169
Phospho-SAPK/JNK (Thr183/Tyr185) Antibody	1:1000	#9251
SAPK/JNK Antibody	1:1000	#9252
CHOP (D46F1) Rabbit mAb	1:1000	#5554
Bax (D2E11) Rabbit mAb	1:1000	#5023
Bcl-2 (D55G8) Rabbit mAb	1:1000	#4223
β-Actin (13E5) Rabbit mAb	1:1000	#4970
Anti-rabbit IgG, HRP-linked Antibody	1:5000	#7074

## Data Availability

The data generated in the present study may be requested from the corresponding author.
